# Immediate Effect on Ground Reaction Forces Induced by Step Training Based on Discrete Skill during Gait in Poststroke Individuals: A Pilot Study

**DOI:** 10.1155/2020/2397374

**Published:** 2020-05-19

**Authors:** Masanori Wakida, Koji Ohata, Yu Hashiguchi, Kimihiko Mori, Kimitaka Hase, Shigehito Yamada

**Affiliations:** ^1^Department of Physical Therapy, Human Health Sciences, Graduate School of Medicine, Kyoto University, Kyoto, Japan; ^2^Department of Rehabilitation, Kansai Medical University Kori Hospital, Osaka, Japan; ^3^Department of Physical Therapy, Faculty of Health Science, Gunma Paz University, Gunma, Japan; ^4^Department of Rehabilitation, Kansai Medical University Hospital, Osaka, Japan

## Abstract

**Methods:**

Twenty-two community-dwelling patients with chronic hemiplegia participated in this study. Eight participants performed only discrete-skill step training during the loading response phase, focusing on paretic hip extension movement (LR group). Another eight performed only discrete-skill step training during the preswing phase, focusing on paretic swing movement (PSw group). The remaining six were trained using both training methods, with at least 6 months in each group to washout the influence of previous training. Therefore, the final number of participants in each group was 14. The braking and propulsive forces of GRFs were measured during gait before and after 30 repetitions of the discrete-skill step training.

**Results:**

Although both groups showed a significant increase in stride length, walking speed was increased only in the LR group. The PSw group showed an increase in braking forces of both sides without any change in propulsion. In the LR group, paretic braking impulse did not change, while nonparetic propulsion increased.

**Conclusion:**

The discrete-skill step training during loading response phase induced an increase in nonparetic propulsion, resulting in increased walking speed. This study provides a clear understanding of immediate effects of the discrete-skill step training in patients with chronic stroke and helps improve interventions in long-term rehabilitation.

## 1. Introduction

Stroke is a leading cause of long-term dysfunctionality in daily living due to motor paralysis, muscle weakness, abnormal muscle tone, and sensory impairments. Although the majority of patients post stroke are able to walk independently, many cannot walk with sufficient speed and endurance for resuming daily activities [1]. Previous studies reported that gait function recovery, 3-6 months after onset, is a plateau in individuals with chronic stroke [2, 3]. Patterson et al. concluded that individuals with a longer recovery period after stroke tend to be more dependent on the nonparalyzed lower limb during gait [4, 5]. Dependence on the nonparetic lower limb can compensate for the paretic lower limb deficit to maintain locomotor functions. However, from a long-term perspective, Patterson et al. also pointed out that this compensation strategy induced several problems, such as loss of bone mineral density of the paretic lower limb and musculoskeletal injury to the nonparetic limb [5]. Therefore, an intervention for improving the paretic limb control is necessary during gait rehabilitation in patients after chronic stroke.

The important walking phase concerned with paretic lower limb deficit is the double-stance phase. The major role of this phase, step-to-step transition, is to control the center of mass (COM) trajectory using ground reaction forces (GRFs) generated from both the paretic and nonparetic limbs [6]. Since this transition appears to be a major determinant of the mechanical work of walking, an improvement in this phase is critically essential to gait rehabilitation [7–9]. In individuals after stroke, paretic propulsion decreases due to the plantar-flexor muscle strength deficit [10]. The push-off power reduction during preswing results in forward propulsion and swing initiation deficits [11, 12]. On the other hand, a longer paretic step and abnormal muscle activation, which are common in patients with stroke, may cause an increase in paretic braking [13, 14]. Turns et al. revealed that the vastus lateralis and biceps femoris correlated positively with paretic braking in early stance phase in severely hemiparetic individuals [13]. Therefore, it is important to improve the GRF control of the paretic lower limb for a smooth step-to-step transition during gait rehabilitation.

Essentially, motor skills are classified into discrete, serial, and continuous [15]. Walking is categorized as a continuous skill, which is a repetition of similar movements. In previous studies, patients after stroke were trained to target a specific gait phase to improve walking performance. Clark et al. reported that increasing step length of the nonparetic limb increased paretic propulsion during late stance [16]. In addition, Genthe et al. reported that gait training using real-time biofeedback on paretic propulsive force improved paretic propulsion and gait biomechanics [17]. On the other hand, patients often face difficulty in changing performance in a specific gait phase. Since walking is a continuous skill, it may be difficult to change control while focusing on a specific gait phase. In such cases, changing to discrete skill, where the movement's start and end are clear, may effectively modify walking performance. Despite the frequent use of step training in actual clinical settings, only few studies examined the role of training by focusing on discrete skills. A clear understanding of immediate effects of the discrete-skill step training in patients with chronic stroke can help improve interventions in long-term rehabilitation.

This pilot study presents a preliminary exploration of long-term interventions in individuals with chronic stroke. We hypothesized that the discrete-skill step training based on loading response and preswing phases of the paretic limb movement results in a faster gait speed immediately by regulating the paretic braking force and propulsion, respectively. This study investigates whether a single session of step training using discrete skills on step-to-step transition can improve walking in individuals with chronic stroke.

## 2. Materials and Methods

### 2.1. Participants

Twenty-two community-dwelling patients with hemiplegia due to chronic stroke participated in this study. All participants could walk independently. The inclusion criteria were as follows: (i) history of a single stroke at least 6 months prior to this study and (ii) the ability to walk independently for at least 5 m without using a cane, with or without ankle-foot orthosis (AFO). The exclusion criteria were as follows: (i) orthopedic diseases affecting measurements and (ii) other neurological diseases such as Parkinsonism and ataxia.

The participants were randomly assigned to each group and trained on the paretic lower limb movement using a discrete skill in one session. Eight participants performed only discrete-skill step training during the loading response phase, focusing on paretic hip extension movement (LR group). The other eight performed only discrete-skill step training during the preswing phase, focusing on paretic swing movement (PSw group). The remaining six were trained using both training methods. In this case, a period of more than 6 months was set up to washout the influence of previous training. Finally, the number of participants added up to 14 in both groups.

This study was approved by the Institutional Ethics Committee. All participants provided written informed consents prior to participation in this study.

### 2.2. Discrete Skill Learning

In this study, we focused on developing discrete skills of the paretic lower limb during two double-stance phases of gait including loading response and preswing.

**Figure 1:**
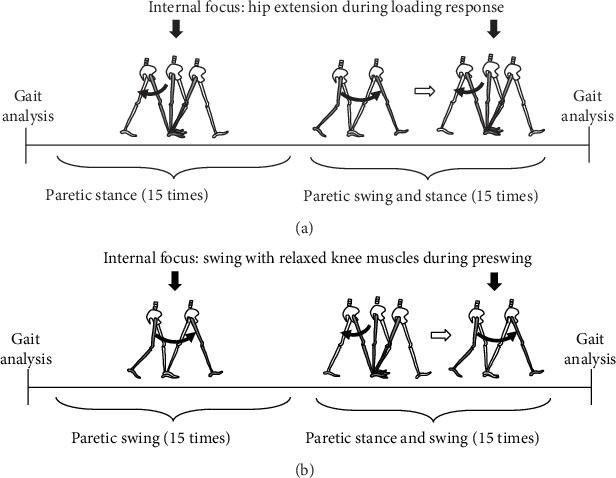
The detailed procedure of discrete-skill step training for (a) the LR group and (b) the PSw group. Paretic and nonparetic limbs were illustrated with black and white color, respectively.

The loading response phase requires weight acceptance and redirection of the COM with the leading limb [9]. In individuals after stroke, paretic braking force tends to increase, so that the COM deceleration in the early stance phase of the paretic limb tends to be excessive [13, 18]. Thus, the LR group was trained to focus on this paretic loading response phase. According to Neptune et al., the hamstrings are muscles that contribute to the COM forward progression during the first stance phase [19]. The training in the LR group consisted of two tasks (Figure 1(a)). In the first task, the participants performed a one-step training. They placed their paretic foot forward and nonparetic foot backward with a stride as large as possible. Then, they were instructed to place their nonparetic limb beyond the paretic foot. This step movement of the nonparetic limb was repeated 15 times. In the second task, the participants performed a two-step training. They placed their nonparetic foot forward and the paretic foot backward and then started the first step of the paretic limb and placed the nonparetic step continuously. This procedure was also repeated 15 times.

The preswing phase is also important since the COM is propelled forward and upward by the propulsion generated on the trailing limb in this phase [9]. In individuals after stroke, the step-to-step transition from the paretic to nonparetic side during preswing is insufficient due to the decrease in paretic propulsion [6]. Thus, swing acceleration is weakened, and the period of preswing tends to be prolonged in hemiplegic gait [12, 20]. Therefore, in the PSw group, the swing movement of the paretic limb during preswing was enhanced using the discrete skill. The PSw group was trained through two tasks (Figure 1(b)). The starting position of the first task was as follows: the nonparetic foot forward and the paretic foot backward. The paretic limb step movement was repeated 15 times to relax the knee. In the second task, the paretic and nonparetic feet were placed forward and backward as the starting position, respectively. The two steps that started from the nonparetic step and the paretic step afterward were performed continuously. This second task was also repeated 15 times to relax the paretic knee during swing phase. If the participant had difficulty in relaxing the paretic knee, instructions were given to quickly move weight to the nonparetic side for shifting the weight away from the paretic limb and smoothing swing initiation.

All participants performed step training by grasping the parallel bar using the nonparetic hand. Those who used an AFO during gait evaluation prior to the step training also used it during step exercise. Prior to the training, one of the two skilled physical therapists instructed verbally and displayed how to perform the step training. In the training, instructions and feedback of the step movement were provided by the therapist. Firstly, the participants in the LR group were given instructions: “Strengthen the hip extension of the paretic limb.” If the therapist observed that the paretic movement was difficult or inadequate, he gave the second instruction: “More forward stepping of the nonparetic limb.” On the other hand, the participants in the PSw group were given the first instruction: “Try to relax the knee of the paretic limb.” Similarly, if the therapist noticed that the paretic movement was performed inadequately or inappropriately, he gave the second instruction: “Immediately transfer the weight toward the nonparetic side.” These second instructions were to facilitate a smooth weight transfer.

### 2.3. Measurements

Gait parameters were measured before and after each step training session. Two pieces of force plates (Kistler Inc., USA) were used to measure GRFs during gait rehabilitation. The force plates were 60 cm long and 40 cm wide. They were arranged side by side along their length in the middle of the walkway (4 m 40 cm). The participants were instructed to walk at a comfortable walking speed without a cane. If some participants were unable to walk without AFO, they were allowed to wear it (they used it during training and postgait evaluation). The GRF data were recorded at a sampling rate of 1,000 Hz during the stance phase on the paretic and nonparetic limbs. Due to the setting of force plates, the GRF data were measured on one side for each trial. The GRF data were collected from more than three successful stance phases of both sides. In addition, the number of steps and the time taken during the 3 m walk were also measured. A time interval of a few minutes was set between the preassessment and training period and between the training period and the postassessment to prevent fatigue. If the participants needed, a further few minutes was given as appropriate.

For the clinical evaluation, the following tests were performed to assess the physical function in both groups: a score on the Fugl-Meyer Assessment (FMA) scale was evaluated for motor function, and the Functional Ambulation Categories (FAC) score was evaluated for walking ability.

### 2.4. Data Processing

GRF data were filtered with a low-pass fourth-order Butterworth filter at 6 Hz forward and backward in time. Peak forces, impulses, and duration time in both braking and propulsive phases were calculated. Each parameter was averaged by the number of measured trials. Peak forces and impulses were normalized according to the participant's body weight. Walking speed and cadence were calculated using the number of steps and the time taken for a 3 m walk. Stride length was also calculated by doubling the average step length.

### 2.5. Statistical Analysis

First, we investigated the normality of all variables using Shapiro-Wilk tests. The differences in GRF parameters, such as peak forces, impulses, and duration of braking and propulsive forces of both sides, were measured before and after the step training and comparatively analyzed using paired *t*-tests for parametric or Wilcoxon signed-rank tests for nonparametric. Similarly, the differences in temporospatial parameters, such as walking speed, stride length, and cadence, were compared using the same tests, as appropriate. Statistical significance was set at *P* < 0.05.

## 3. Results

Two participants in the LR group and one participant in the PSw group were excluded from the analysis because it was difficult for them to walk by placing the affected limb properly on the force plates. Therefore, the enrolled participants in this study finally were 12 in the LR group and 13 in the PSw group. Six participants in the LR group and ten participants in the PSw group were not able to walk without AFO, so they were allowed to use the AFO during step training and gait evaluation.

Table 1 shows the participants' physical characteristics. The mean duration from the stroke onset was 5.7 (standard deviation: 4.5) years in the LR group and 8.4 (4.7) years in the PSw group. The motor function (FMA) of the paretic lower limb was 22.5 (4.6) points and 22.7 (5.2) points, in the LR group and the PSw group, respectively. The walking speeds in the LR and PSw groups were 0.67 (0.30) m/s and 0.54 (0.20) m/s, respectively.

**Figure 2:**
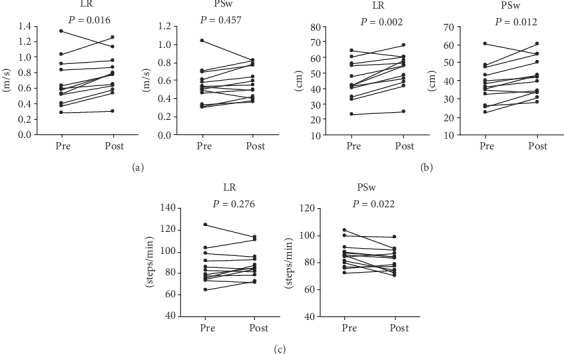
Individual changes in (a) gait speed, (b) stride length, and (c) cadence before and after training in both groups.

As immediate effects on the LR group, stride length and walking speed increased significantly after the training (Table 2, Figure 2). Among the GRF parameters, paretic peak braking force significantly increased (*P* = 0.038), whereas impulse and duration time did not change significantly (*P* = 0.978, *P* = 0.120, respectively). Nonparetic propulsive forces increased significantly (peak: *P* = 0.003, impulse: *P* = 0.019). Simultaneously, the duration time of the nonparetic propulsive phase decreased significantly. While nonparetic braking forces increased significantly (peak: *P* = 0.006, impulse: *P* = 0.012), paretic propulsive forces did not change significantly but tended to increase (peak: *P* = 0.077, impulse: *P* = 0.060).

Regarding immediate effects on the PSw group, stride length increased, and cadence decreased significantly (Table 2, Figure 2). However, walking speed did not change after training. Among the GRF parameters, only braking forces (peak and impulse) of both limbs increased significantly. The duration of the nonparetic braking phase increased significantly as well.

## 4. Discussion

This study revealed that the single discrete-skill step training session could immediately change paretic and nonparetic GRFs while performing a continuous skill such as walking. The findings of this study can be beneficial for gait rehabilitation in patients with gait dysfunction due to chronic stroke.

### 4.1. LR Group

The stride length and walking speed immediately increased in patients of the LR group after the repetitive step training that emphasized on the paretic hip extension during loading response. In general, the impulses of anteroposterior GRFs are expected to increase as the stride length and walking speed increase [21, 22]. In this study, the paretic peak braking force, nonparetic peak propulsive force, and impulse increased significantly as the walking speed increased. Most remarkably, these changes in GRFs corresponded to the paretic loading response phase during walking, suggesting that the discrete-skill step training induced task-specific changes in GRFs. In a previous study, stroke survivors relied on their nonparetic propulsive forces to increase walking speed [21]. However, if paretic braking forces were not controlled adequately, their nonparetic propulsion would have been limited as well. Therefore, nonparetic propulsion was increased due to the improvement in the control of paretic braking forces in the LR group.

Nonparetic propulsion increased significantly with peak force and impulse in this study. However, paretic braking force increased only in peak force, not in impulse. It is noteworthy that there was a difference between changes in peak force and impulse in paretic braking forces. The increase in peak braking force of the paretic leading limb suggests that the paretic limb could receive a greater propulsive force from the nonparetic limb as walking speed increases. On the other hand, no change in paretic braking impulse would indicate another reason. Turns et al. reported that paretic braking impulse increased as compared to the nonparetic limb in individuals with severe hemiparesis [13]. This excessive braking force on the paretic limb possibly causes greater deceleration of COM during the stance phase. That is, patients in the LR group were deemed able to absorb the shock instantly during the early stance phase of the paretic limb, resulting in an increased peak force despite no change in impulse of paretic braking force. This result is consistent with our hypothesis that the discrete-skill step training during paretic loading response leads to an increase in gait speed immediately by regulating paretic braking force.

Neptune et al. showed that the hamstring muscles accelerated the trunk forward at the beginning of stance [19]. Since the hamstrings are the main muscles of hip extension during the loading response, this single session of repetitive step training enhanced the paretic hip extension. We postulated that the forward acceleration of COM by hip extensor muscles increases trailing limb angle (hip extension) at the late stance phase and increases paretic propulsion. However, since paretic propulsion is a critical problem of hemiplegic gait [23], it did not change in the LR group. The reason for this result can possibly be attributed to the single training session. Therefore, a long-term intervention based on repetitive discrete skill-based step training might be able to improve paretic propulsion.

### 4.2. PSw Group

The stride length of PSw group patients increased, although their cadence was decreased without changes in walking speed. A decreased cadence indicated a prolonged gait cycle. Therefore, although the stride length was increased, walking speed remained unchanged due to the prolonged gait cycle. Only braking forces (peak and impulse) of both limbs increased along with the increase in stride length. Conversely, the propulsive forces of both limbs did not change.

Previous studies on walking simulations reported that the triceps surae muscles contribute to the propulsion and swing of the lower limb [11]. The propulsive force in the late stance phase provides push-off power and leads to swing acceleration with knee flexion during the swing phase simultaneously [12, 20, 24]. However, it is often difficult in individuals after stroke to generate paretic propulsion due to the impaired triceps surae muscles [13, 23, 25, 26]. Therefore, reduced paretic propulsion after stroke is one of the causes of inadequate knee motion during the swing phase. In addition, a simulation study on normal gait revealed that the gastrocnemius muscle is the largest contributor to peak knee flexion velocity during preswing, while the vasti and rectus femoris muscles decrease this velocity [27]. Therefore, paretic swing movement was enhanced with consciousness of relaxing the knee in the PSw group. Since paretic propulsion did not change in the PSw group, it was difficult for patients to walk with an immediate increase in the output of the triceps surae muscles because of the single training session. A previous study reported a negative correlation between paretic propulsion and the hip joint moment [26]. Thus, the hip flexor moment compensates for the push-off weakness due to impaired plantar-flexor muscles in a typical hemiplegic gait [28]. Therefore, the PSw group patients may compensate for the push-off weakness using their hip flexor muscles.

The training in the PSw group emphasized on knee flexion during paretic swing phase. However, the difficulty of paretic swing control may slow down the swing motion and lead to a prolonged gait cycle after training. In addition, we ascribed that the step length increased due to the increase in paretic swing movement, resulting in an increase in paretic braking force. Previous studies reported that the iliopsoas is also a contributor muscle to the knee flexion during preswing [19, 27]. Regarding the hip pull-off, weight transfer to the leading limb should be performed smoothly, not delayed because the release timing of lengthening the hip flexor is critical to offloading [29]. Therefore, to make it easier to swing the paretic limb, we instructed the patients to move weight toward the nonparetic side quickly during the preswing phase of the paretic limb. As a result, there is a possibility that the nonparetic braking force also increases. These results suggest that increasing paretic propulsion by training the plantar-flexor muscles is an important strategy for improving the step-to-step transition during the preswing phase in individuals after stroke.

### 4.3. Clinical Implication

If clinicians want to increase the patient's walking speed immediately, the step training with consciousness of the paretic hip extension during loading response may be beneficial. On the other hand, although propulsive force is generated during the preswing phase, it may be difficult to improve walking speed immediately by step training with consciousness of paretic swing movement.

There are some limitations to this study. First, the effects of step training using a discrete skill were not compared with another training using a continuous skill. In the future, it is necessary to clarify the effects of step training using discrete skill by comparison with a step training session based on using continuous skill. Second, more detailed kinetic and kinematic parameters, such as step length of both limbs and joint motion, could not be measured because of environmental hindrances. Further investigation is needed to reveal the effects of discrete-skill step training using more detailed gait parameters.

## 5. Conclusions

The current study revealed that a single session of repetitive step training using the discrete skill could change gait performance immediately in individuals after chronic stroke. Notably, discrete-skill step training, which focuses on paretic hip extension movement during loading response, immediately increases walking speed because of the increased nonparetic propulsion by regulating paretic braking force. On the other hand, the step training that focused on paretic swing movement during preswing immediately increased stride length and decreased cadence. The participants in this group immediately increased only braking forces of both limbs with no change in walking speed. These findings suggest that step training may be useful as an explicit learning to change GRFs during gait.

## Figures and Tables

**Table 1:** Physical function, clinical measurements, and gait parameters of the LR and PSw groups before training.

	LR (*n* = 12)	PSw (*n* = 13)	LR-only participants (*n* = 6)	PSw-only participants (*n* = 7)	Both groups' participants at LR training (*n* = 6)	Both groups' participants at PSw training (*n* = 6)
Age	44.5 (20.2)	53.9 (14.1)	36.7 (20.6)	53.7 (9.7)	52.3 (18.1)	53.2 (18.1)
Sex (male/female)	6/6	11/2	1/5	6/1	1/5	
Type (ischemia/hemorrhage)	4/8	3/10	2/4	1/6	2/4	
Hemiparetic side (right/left)	7/5	9/4	3/3	5/2	4/2	
Time since stroke (years)	5.7 (4.5)	8.4 (4.7)	3.6 (1.6)	7.8 (4.0)	7.9 (5.5)	8.6 (5.6)
Clinical measurement						
Fugl-Meyer Assessment (motor)	22.5 (4.6)	22.7 (5.2)	21.5 (5.0)	21.9 (6.6)	23.5 (4.4)	23.7 (3.2)
Functional Ambulation Categories (5/4/3)	9/1/2	12/1/0	4/1/1	7/0/0	5/0/1	5/1/0
Gait						
Speed (m/s)	0.67 (0.30)	0.54 (0.20)	0.67 (0.38)	0.54 (0.08)	0.66 (0.25)	0.55 (0.29)
Stride length (m)	0.45 (0.12)	0.38 (0.10)	0.44 (0.15)	0.38 (0.05)	0.45 (0.10)	0.38 (0.15)
Cadence (steps/min)	85.7 (16.3)	85.1 (9.0)	85.7 (20.9)	85.5 (3.6)	85.7 (12.2)	84.6 (13.3)

Values expressed as mean (standard deviation).

**Table 2:** Immediate changes in gait parameters before and after a single training session of the LR and PSw groups.

	LR (*n* = 12)		*P* value	PSw (*n* = 13)		*P* value
	Pre	Post		Pre	Post	
Speed (m/s)	0.67 (0.30)	0.77 (0.26)	0.016∗	0.54 (0.20)	0.58 (0.17)	0.457
Stride length (m)	0.45 (0.12)	0.51 (0.11)	0.002∗∗	0.38 (0.10)	0.42 (0.10)	0.012∗
Cadence (steps/min)	85.7 (16.3)	87.8 (13.1)	0.276	85.1 (9.0)	81.5 (8.2)	0.022∗
GRF parameters						
Paretic braking						
Peak force (%BW)	14.2 (5.1)	15.6 (5.6)	0.038∗	11.0 (3.6)	13.0 (4.8)	0.009∗∗
Impulse (%BW·s)	2.8 (0.9)	2.8 (0.8)	0.978	2.1 (0.7)	2.6 (0.9)	0.004∗∗
Duration (s)	0.44 (0.06)	0.41 (0.06)	0.120	0.44 (0.16)	0.44 (0.08)	0.962
Paretic propulsion						
Peak force (%BW)	7.6 (4.6)	9.4 (5.3)	0.077	5.3 (3.7)	5.7 (3.3)	0.501
Impulse (%BW·s)	1.6 (1.0)	2.0 (1.0)	0.060	1.1 (0.9)	1.2 (0.9)	0.602
Duration (s)	0.36 (0.12)	0.38 (0.10)	0.481	0.32 (0.12)	0.31 (0.13)	0.594
Nonparetic braking						
Peak force (%BW)	13.1 (6.1)	16.5 (5.8)	0.006∗∗	12.6 (4.1)	14.2 (2.8)	0.036∗
Impulse (%BW·s)	2.5 (1.1)	3.1 (1.1)	0.012∗	2.3 (1.0)	2.8 (1.0)	0.000^†^
Duration (s)	0.43 (0.15)	0.43 (0.10)	0.519	0.43 (0.12)	0.49 (0.17)	0.028∗
Nonparetic propulsion						
Peak force (%BW)	16.9 (7.3)	21.5 (7.1)	0.003∗∗	12.7 (4.7)	14.2 (5.4)	0.246
Impulse (%BW·s)	4.0 (1.2)	4.6 (1.2)	0.019∗	3.5 (1.2)	3.7 (1.3)	0.413
Duration (s)	0.62 (0.17)	0.55 (0.13)	0.011∗	0.64 (0.14)	0.60 (0.11)	0.110

∗*P* < 0.05, ∗∗*P* < 0.01, and ^†^*P* < 0.001. Values expressed as mean (standard deviation). GRF: ground reaction force; %BW: percent body weight.

## Data Availability

The data used to support the findings of this study are available from the corresponding author upon request.
